# Quartz crystal microbalance–based aptasensor integrated with magnetic pre-concentration system for detection of *Listeria monocytogenes* in food samples

**DOI:** 10.1007/s00604-024-06307-2

**Published:** 2024-04-03

**Authors:** Fatma Beyazit, Mehmet Yakup Arica, Ilkay Acikgoz-Erkaya, Cengiz Ozalp, Gulay Bayramoglu

**Affiliations:** 1 Department of Obstetrics and Gynecology, Faculty of Medicine, Çanakkale Onsekiz Mart University, Çanakkale, Turkey; 2 Biochemical Processing and Biomaterial Research Laboratory, Gazi University, 06500 Teknikokullar, Ankara, Turkey; 3 Department of Environmental Science, Faculty of Engineering and Architecture, Ahi Evran University, Kırsehir, Turkey; 4 Department of Medical Biology, School of Medicine, Atilim University, Ankara, Turkey; 5 Department of Chemistry, Faculty of Sciences, Gazi University, 06500 Teknikokullar, Ankara, Turkey

**Keywords:** Magnetic particles, Pre-concentration, Aptasensor, Quartz crystal microbalance, *Listeria monocytogenes* detection

## Abstract

A fast and accurate identification of *Listeria monocytogenes.* A new quartz crystal microbalance (QCM) aptasensor was designed for the specific and rapid detection of *L. monocytogenes*. Before detection of the target bacterium from samples in the QCM aptasensor, a magnetic pre-enrichment system was used to eliminate any contaminant in the samples. The prepared magnetic system was characterized using ATR-FTIR, SEM, VSM, BET, and analytical methods. The saturation magnetization values of the Fe_3_O_4_, Fe_3_O_4_@PDA, and Fe_3_O_4_@PDA@DAPEG particles were 57.2, 40.8, and 36.4 emu/g, respectively. The same aptamer was also immobilized on the QCM crystal integrated into QCM flow cell and utilized to quantitatively detect *L. monocytogenes* cells from the samples. It was found that a specific aptamer-magnetic pre-concentration system efficiently captured *L. monocytogenes* cells in a short time (approximately 10 min). The Fe_3_O_4_@PDA@DA-PEG-Apt particles provided selective isolation of *L. monocytogenes* from the bacteria-spiked media up to 91.8%. The immobilized aptamer content of the magnetic particles was 5834 µg/g using 500 ng Apt/mL. The QCM aptasensor showed a very high range of analytical performance to the target bacterium from 1.0 × 10^2^ and 1.0 × 10^7^ CFU/mL. The limit of detection (LOD) and limit of quantitation (LOQ) were 148 and 448 CFU/mL, respectively, from the feeding of the QCM aptasensor flow cell with the eluent of the magnetic pre-concentration system. The reproducibility of the aptasensor was more than 95%. The aptasensor was very specific to *L. monocytogenes* compared to the other *Listeria species* (i.e., *L*. *ivanovii*, *L*. *innocua*, and* L. seeligeri*) or other tested bacteria such as *Staphylococcus aureus*, *Escherichia coli*, and *Bacillus subtilis*. The QCM aptasensor was regenerated with NaOH solution, and the system was reused many times.

**Graphical Abstract:**

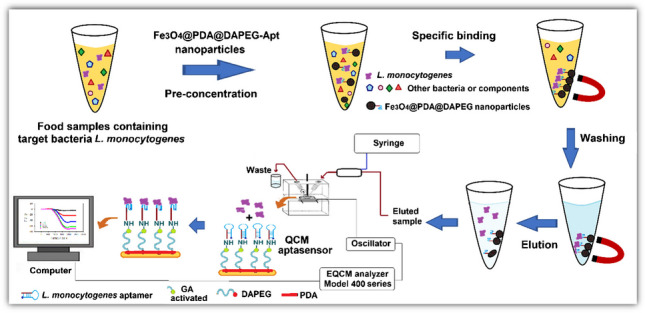

**Supplementary Information:**

The online version contains supplementary material available at 10.1007/s00604-024-06307-2.

## Introduction

Foodborne diseases are caused by consuming food contaminated by pathogens, viruses, parasites, and other pollutants. The most important foodborne pathogens are *L. monocytogenes*, *E. coli*, *S. enterica* serovars, *S. aureus*, *B. cereus*, *C. sakazakii*, *Campylobacter* spp., *Vibrio* spp., *Shigella* spp., *Y. enterocolitica*, *C. botulinum*, and *C. perfringens* [1]. The rapid determination of these pathogens possibly found in the food is very important to provide food safety. Among these bacteria, *Listeria monocytogenes*, a gram-positive intracellular rod-shaped bacterium, is one of the most known foodborne pathogens in the world. It is catalase-positive and has beta-hemolytic activity when grown on blood agar [2]. *Listeria* spp. are capable of growing well even at refrigeration temperatures from 2.0 to 8 °C, thus causing contamination of ready-to-eat foods with a considerable number of bacteria [3]. Generally, infection with *L. monocytogenes* is not restricted to sepsis, meningitis, encephalitis, spontaneous abortion, or fever and self-limiting gastroenteritis in a healthy adult. People at the most risk for *L. monocytogenes* are pregnant females, infants, immunocompromised individuals, and the elderly [4–6]. *Listeria monocytogenes* causes *listeriosis* with a 20–30% death rate. *Listeria monocytogenes* can withstand the most common stress levels, such as high salinity, acidity, refrigeration temperatures, and low water activity. It is also known as a hazardous pathogen bacterium and poses a contamination risk to ready-to-eat food. Therefore, its rapid detection from food samples is essential [5, 7].

For the determination of *L. monocytogenes*, various technologies have been reported, such as immune-magnetic separation, ELISA-based assays, immune-chromatographic technology, DNA hybridization, and PCR method [8–10]. All these methods need a selective enrichment culture and a cultivation time of at least 24 h. Then, selective nutrient agars and further confirmatory tests are necessary for the detection of the target bacterium. Specific PCR-based assays could detect *L. monocytogenes* within 30 h; therefore, the development of rapid isolation is an active objective in the research areas. In previous studies, many methods have been reported for quantitatively determining pathogens in the food sample using QCM and SPR sensors [11–16]. In the QCM sensor system, the determination of target bacteria mainly relies on a decrease in the frequency change linearly proportional to the attached mass on the functionalized QCM crystal, known as the Sauerbrey relation [17–20]. In this method, a specific recognition molecule (e.g., aptamer or antibody) is immobilized on the sensor chip surface to recognize the target bacterium with specific interaction. Thus, non-covalent interactions between the target analyte and the recognition molecule on the QCM chip surface can cause a detectable frequency change even at any concentration [21–24]. By using this method, specific interactions between the target bacterium and the recognition molecule on the QCM chip surface could be created for the quick determination of foodborne pathogens in food samples. Although many methods have been reported to detect pathogens in food samples, nonspecific interactions hinder real-life applications of sensor systems [19, 25–27]. Therefore, the magnetic pre-enrichment technique has been used to prevent the interaction of impurities with the QCM system for the isolation of protein, cells, and fine chemicals from complex solutions without using expensive laboratory equipment. The unique, exciting property of the magnetic adsorbent system is that after being laden with the target analyte, the magnetic adsorbent can be easily separated from the medium by implementing an external magnet [28–38]. In the previously reported studies, a new strategy has been applied for the rapid determination of pathogens in food samples by combining the magnetic pre-concentration system with the QCM sensor system [39–42]. This method may be a reliable approach for isolating and determining pathogens from complex food samples due to the two-time selection of target bacterium [18, 40, 41]. Recently, specific aptamer ligands have been used to identify target bacteria using a biosensor system [18, 21]. These ligands are short-chain DNA and RNA sequences and are called aptamer ligands. These ligands have been proposed and utilized for specific interaction and recognition with their target with a specific high affinity. Aptamers are biological recognition molecules and can be chosen from complex nucleic acid libraries by the combinatorial chemistry method known as systematic evolution of ligands by exponential enrichment (SELEX) [17, 18, 21]. Thus, aptamer ligands can be selected in vitro for their specific interaction with their target molecule [17, 18]. Furthermore, aptamer ligands have several advantages over traditional antibody ligands [12, 18]; for example, they have better stability, smaller sizes, and easy chemical modifications and can be synthesized in laboratory environments. These advantages ensure the aptamer ligand is a well-targeted and target-specific interaction molecule in the designed sensor system. Various aptamer ligands have been effectively immobilized on the differently prepared composite magnetic materials and used for the isolation and detection of target bacteria from food samples [13, 21, 42–44].

In this study, a specific recognition aptamer for *L. monocytogenes* was selected using the SELEX method. A new method was proposed by combining aptamer-based magnetic pre-concentration system with a QCM sensor for rapid and sensitive detection of *L. monocytogenes* in the food samples. The traditional methods for the detection of *L. monocytogenes* rely on cell culture, enrichment, isolation, biochemical analysis, and immunological identification. These methods are time-consuming, expensive, and high-cost. Therefore, a sentient binary system was presented as an alternative method for the rapid detection and quantification of *L. monocytogenes* from solid or liquid food samples. To achieve this goal, magnetic particles and QCM chips were grafted sequentially with polydopamine and then with diamino-polyethylene glycol without using any activating agent; then, glutaraldehyde was used as a linker to covalent attachment of the selected *L. monocytogenes*–specific aptamer. The eluent from the magnetic pre-concentration system was contacted by the QCM sensor to produce the final signal as a result of the interaction of the *L. monocytogenes* to the QCM chip surface. Furthermore, the selectivity of the QCM aptasensor was studied by evaluating the sensing of its efficiency against other bacteria species, such as three different *Listeria* spp., *E. coli*, *S. aureus*, and *B. subtilis*.

## Materials and methods

### Materials

Diamino-polyethylene glycol, dopamine hydrochloride, Trizma® hydrochloride, glutaraldehyde, glycerol, and diiodomethane were supplied from Sigma-Aldrich. *Listeria ivanovii* ATCC 19119, *Listeria innocua* ATCC 33090 and *Listeria seeligeri* ATCC 35967, *Listeria monocytogenes* (ATCC 7644), *Escherichia coli* (ATCC 12435), and *Staphylococcus aureus* (ATCC 43300) were purchased from the American Type Culture Collection (Rockville, Md.). All other chemicals were of analytical grade and purchased from Merck AG (Darmstadt, Germany). QCM crystal (15-mm diameter with polished Au electrodes diameter = 5.0 mm, thickness = 1000Å, 10.0-MHz AT-cut piezoelectric quartz wafer for EQCM) was obtained from CH Instruments, Texas, USA.

### Aptamer selection

The specific binding aptamer for pathogens was selected using SELEX method as described previously [12, 18]. *L. monocytogenes* (ATCC 7644) cells were used in the SELEX study. *L. monocytogenes* and 100 nmol SELEX library were transferred in 1.0 mL PBS and incubated for 2.0 h at 25 °C. *L. monocytogenes* cells with interacted DNA sequences from the library were removed from the medium by centrifugation at 4000 rpm for 15 min. After the cells were washed with PBS, the bounded DNA sequences were eluted with NaOH solution (100 mmol/L) and amplified by PCR (95 °C, 3.0 min), 35× (95 °C, 1.0 min; 61 °C, 1.0 min; 72 °C, 1.0 min, 72 °C, 5.0 min). The amplification products were analyzed with 4% (w/v) agarose gel electrophoresis for determination of correct size, then streptavidin-coated magnetic nanoparticles (Dynabeads, Invitrogen) were used to obtain single-stranded library sequences to capture biotin-labeled strands. Unlabeled single-stranded sequences were eluted with NaOH solution (100 mM). Before starting the next cycle, the negative selection mixture, namely, *L. ivanovii* ATCC 19119, *L. innocua* ATCC 33090, and *L. seeligeri* ATCC 35967, was contacted for 1.0 h, and the cell mixture was removed by centrifugation. The supernatant obtained after the interaction (i.e., the DNA sequences not binding to negative strains) was used in the previous *L. monocytogenes* selection. The binding amount of DNA sequences from library members to *L. monocytogenes* was evaluated at the end of each cycle. For this purpose, the PCR product obtained in each SELEX cycle was amplified with a 5′-fluorescently labeled primer and investigated by measuring the fraction of DNA library members to the *L. monocytogenes*. After each cycle, single-stranded versions of fluorescently labeled PCR products were incubated with target bacterium or non-target bacteria and washed twice with buffer solution, and quantitative analysis of the remaining aptamer sequences on the cell was calculated by fluorescent spectroscopy. At the end of the enrichment analysis, it was observed that the binding rates did not increase after the 10th cycle. The 10^th^-cycle sequences were obtained by next-generation sequencing (iSeq100, Illumina). For this purpose, 10th-cycle PCR products were prepared for sequencing following Nextera v2 (Illumina) kit procedures, and the aptamer candidates were characterized for kinetic parameters.

### Synthesis of Fe_3_O_4_ nanoparticles

Fe_3_O_4_ nanoparticles were synthesized as described previously [45, 46]. Briefly, FeCl_3_·6H_2_O (5.6 g) and FeSO_4_.7H_2_O (3.0 g) were transferred in a round-bottomed flask (1000 mL) and 400 mL of deionized water was added. The mixture was stirred using an overhead mixer for 30 min. The temperature was raised to 70 °C, and 100 mL of NH_4_Cl solution (25%) was added using a dropping funnel. The mixture was refluxed for 2.0 h under a nitrogen atmosphere. Finally, the medium temperature was elevated to 90 °C and refluxed for about 1.0 h. The synthesized Fe_3_O_4_ nanoparticles were separated from the medium using an external magnet and cleaned with distilled water.

### Preparation aptamer immobilized magnetic nanoparticles

As described previously, the Fe_3_O_4_ particles were grafted with poly(dopamine) [15, 47]. Briefly, 5.0 g of magnetic nanoparticle was transferred into Tris-HCl buffer solution (10 mmol/L, pH 8.5, 250 mL) containing ethyl alcohol (75 mL) and 800 mg dopamineˑ HCl. The mixture was stirred at 60 °C for 18 h. Then, polydopamine-coated Fe_3_O_4_@PDA particles were separated using a permanent magnet. They were washed with ethyl alcohol (200 mL) and purified water. They were dried under reduced pressure at 45 °C. The Fe_3_O_4_@PDA particles were coated with diamino-polyethylene glycol (DA-PEG) and characterized as described earlier [48]. For aptamer immobilization, the Fe_3_O_4_@PDA@DA-PEG particles were reacted with glutaraldehyde (1.0%, 100 mL) as described previously [49]. These activated particles were used for the immobilization of *L. monocytogenes-*specific aptamers. For this, *L. monocytogenes*–specific aptamer ligand 500 ng/mL in Tris-HCl buffer (25 mL) at pH 8.0 was incubated with the activated magnetic particles at 45 °C. The amount of immobilized aptamer on Fe_3_O_4_@PDA@DA-PEG-GA particles was determined spectrophotometrically. The absorbance of these solutions was obtained at 265 nm using a UV-Vis NanoDrop 2000 (NanoDrop Products, Wilmington, USA).

### Characterization of magnetic beads

The grafting percentage (GP) of the polymer onto Fe_3_O_4_ was determined by calculating the percentage increase in weight using the following equation:

1 $$\mathrm{GP }= [({{\text{M}}}_{{\text{g}}} - {{\text{M}}}_{0}) / {{\text{M}}}_{0}] \times 100$$

where *M*_0_ and *M*g are the weights of the Fe_3_O_4_ particles before and after grafting, respectively [50]. The determination of water content of the Fe_3_O_4_@PDA@DA-PEG-GA particles was studied, as reported earlier [50]. The equilibrium water content was calculated using the following equation:

2 $$\mathrm{Equilibrium\, water\, content}: (\mathrm{\%}) = [({{\text{M}}}_{{\text{s}}}- {{\text{M}}}_{{\text{d}}}) / {{\text{M}}}_{{\text{d}}})] \times 100$$

where *M*_s_ and *M*_d_ are the swelled and dry masses of the particles, respectively. The magnetic properties of the Fe_3_O_4_ and Fe_3_O_4_@PDA@DA-PEG-Apt particles were determined with a vibrating-sample magnetometer (VSM; Model 155, Digital Measurement System Inc., Westwood, MA, USA) at room temperature. The specific surface areas of the Fe_3_O_4_ and Fe_3_O_4_@PDA@DAPEG-Apt particles were determined using the BET (Brunauer, Emmett, and Teller) method using a Quantachrome Nova 2200E, USA. Thermal gravimetric analyses (TGA/TGS) of the Fe_3_O_4_ and Fe_3_O_4_@PDA@DAPEG-Apt particles were studied under nitrogen flow in the temperature range of 25–800 °C and at a heating rate of 10 °C/min. It was performed with a thermal analyzer (NETZSCH STA 449 F3 Jupiter DTA/TG).

### Preparation of QCM crystal for detection of *L. monocytogenes*

The QCM crystal was placed between two O-rings into a flow cell. The gold-plated quartz crystal of the QCM sensor was modified by coating two-layer polymer layers as described above. Briefly, the crystal was cleaned with piranha solution (30% H_2_O_2_:H_2_SO_4_/1:3) for 15 min, washed with ethanol and water, and dried in a vacuum oven. The poly(dopamine) (PDA) layer on the surface of the QCM crystal (Chip) was formed by contacting with Tris-HCl solution containing 20 mmol/L dopamine for 16 h, and the self-assembled layer was synthesized without the need for any activation agent. The PDA-coated crystal surface was washed sequentially with ethanol and distilled water, and this coating provided a reactive surface for further modification of the surface of the QCM crystal. Then, the PDA-coated QCM crystal surface was grafted with DA-PEG. For this, Tris-HCl buffer (10 mM, pH 8.5) and DA-PEG solution of known concentration were contacted with the Chip@PDA for 8.0 h at 65 °C. At the end of this treatment step, the DA-PEG coated crystal (i.e., Chip@PDA@DA-PEG) was washed with ethanol and purified water. The frequency changes resulting from contacting one side of the quartz crystal in the flow cell with the dopamine, DAPEG, or aptamer solutions at a fixed flow rate (50 µL/s) were monitored. The immobilization of *L. monocytogenes*–specific aptamer on the Chip@PDA@DA-PEG was realized after glutaraldehyde activation. The Chip@PDA@DA-PEG surface was activated in glutaraldehyde solution (0.1%; pH 7.4) at 60 °C for 6 h. Frequency changes after this activation process were also recorded. Then, the activated (Chip@PDA@DA-PEG-GA) was removed from the reaction medium and washed sequentially with water, acetic acid solution, and phosphate buffer (pH 7.4). Then, the Chip@PDA@DA-PEG-GA surface was contacted with a solution containing *L. monocytogenes*–specific aptamer in Tris-HCl buffer (pH 8.0). The immobilization of the amine-labeled *L. monocytogenes* aptamer was performed at 45 °C for 6.0 h. After this period, the Chip@PDA@DA-PEG-Apt crystal was removed from the flow cell, washed with NaCl solution, and then with buffer solution to remove unbound or physically attached aptamer ligand.

In these experimental studies, the used quartz crystal micro-balance system (Model CHI 400A, CH Instruments, Texas, USA) consists of a continuous flow cell, oscillator, and analyzer. The as-prepared (Chip@PDA@DA-PEG-Apt) was placed in the flow cell holder, and then, it was used to perform target bacterium analyses by connecting the oscillator and analyzer. The samples were injected into the flow cell using an injection pump, and the flow rate was adjusted at a predetermined rate. The device was controlled by a computer and operated under the Windows XP operating system. Calculations were made according to the Sauerbrey equation:

3 $$\mathrm{\Delta F }= -{{2{\text{F}}}_{0}}^{2}\mathrm{ \Delta m }/ [\mathrm{A }{(\mathrm{\mu \rho q})}^{0.5}]$$

Here, *F*_0_ is the resonance frequency of the crystal in its ground state; Δm is the mass change at the surface; *A* is the area of the gold-coated crystal; *ρ*_q_ is the density of the crystal (2.684 × 10^9^ ng/cm^3^); and *µ* represents the shear modulus of quartz (2.947 × 1020 ng/cm/s^2^). Δm/A denotes the increase in mass per unit area (ng/cm^2^). The mass change (Δm) of the aptasensor corresponding to this frequency shift was calculated by measuring the target bacteria *L. monocytogenes*’ cells in the medium and/or real samples before and after the analysis.

The frequency changes in the QCM sensor were recorded to detect different amounts of chemicals interacting with the sensor surface after passing each sample through the flow cell and used to determine the amount of mass deposited on the crystal surface upon the addition of polydopamine, DAPEG, aptamer, or bacteria. According to the QCM system manufacturer’s manual (CH Instruments), a net change of 1.0 Hz corresponds to a mass change of 1.34 ng over a crystal surface area of 0.196 cm^2^.

The contact angle measurements were made to determine changes on the surface of QCM chips after each modification step. The contact angle values of the crystal, Cry@PDA, Chip@PDA@DA-PEG, Chip@PDA@DA-PEG-GA, Chip@PDA@DA-PEG-Apt, and *L. monocytogenes* bacterium–attached crystal (i.e., Chip@PDA@DA-PEG-Apt) surfaces were determined. Contact angle values of the samples were studied using different test liquids (water, glycerol, and diiodomethane) using the sessile drop method at 25 °C. A digital optical contact angle meter Phoenix 150 (Surface Electro Optics, Korea) was used in the studies. The right and left contact angles of each drop dropped on the sensor surface, and the drop size parameters were automatically calculated from the digital image and measured as a function of time at 10-s intervals. The average contact angle value was calculated by taking the average of at least six measurements [18]. Using the determined contact angle values, the surface energies and surface energy parameters were calculated using van Oss method [51]. The concept of wetting a solid surface with a liquid and the contact angle (θ) formulated by Young is given by the following equation.4where *ɣ*_l_ is the surface energy of the liquid, *ɣ*_sl_ is the interfacial energy of the solid/liquid interface, and *ɣ*_s_ is the surface energy of the solid.

### Bacterial cultures

*L*. *monocytogenes* reference strain (ATCC 7644), *L*. *ivanovii* (ATCC 19119), *L. innocua* (ATCC 33090), *L. seeligeri* (ATCC 35957), *S. aureus*, *E. coli*, and *B. subtilis* were grown overnight at 37 °C in Tryptone Soy Broth (TSB). The desired concentrations of cultures were prepared by serially diluting with a physiological buffer solution (PBS, pH 7.4). The samples were grown on a Tryptone Soy Agar plate for colony-counting experiments (TSA, Oxoid CM0131B). The Petri plates were incubated at 37 °C for 18 h under aseptic conditions using standard microbiology techniques. Bacterial cultures were prepared from glycerol stocks frozen at −80 °C, and the single colony-forming units (CFUs) were recorded.

In the preparation of chicken meat and milk growth media, 10 g of chicken meat was added into 90 mL of PBS and homogenized for 2.0 min. After centrifugation, this mixture at 1000 rpm for 15 min, the chicken meat supernatant (10 mL) was added to the growth medium of *L. monocytogenes* as described above. The milk-containing medium was prepared by adding 10 mL of milk to the above growth medium.

### Statistical analysis

The experimental studies were triplicated, the data average was reported, and both material preparations and bacterial counts in CFU/mL were included. All of the obtained results were reported as mean values ± standard deviation (SD).

## Results and discussion

### Characterization studies of the Fe_3_O_4_@PDA@DAPEG-Apt particles

**Fig. 1:**
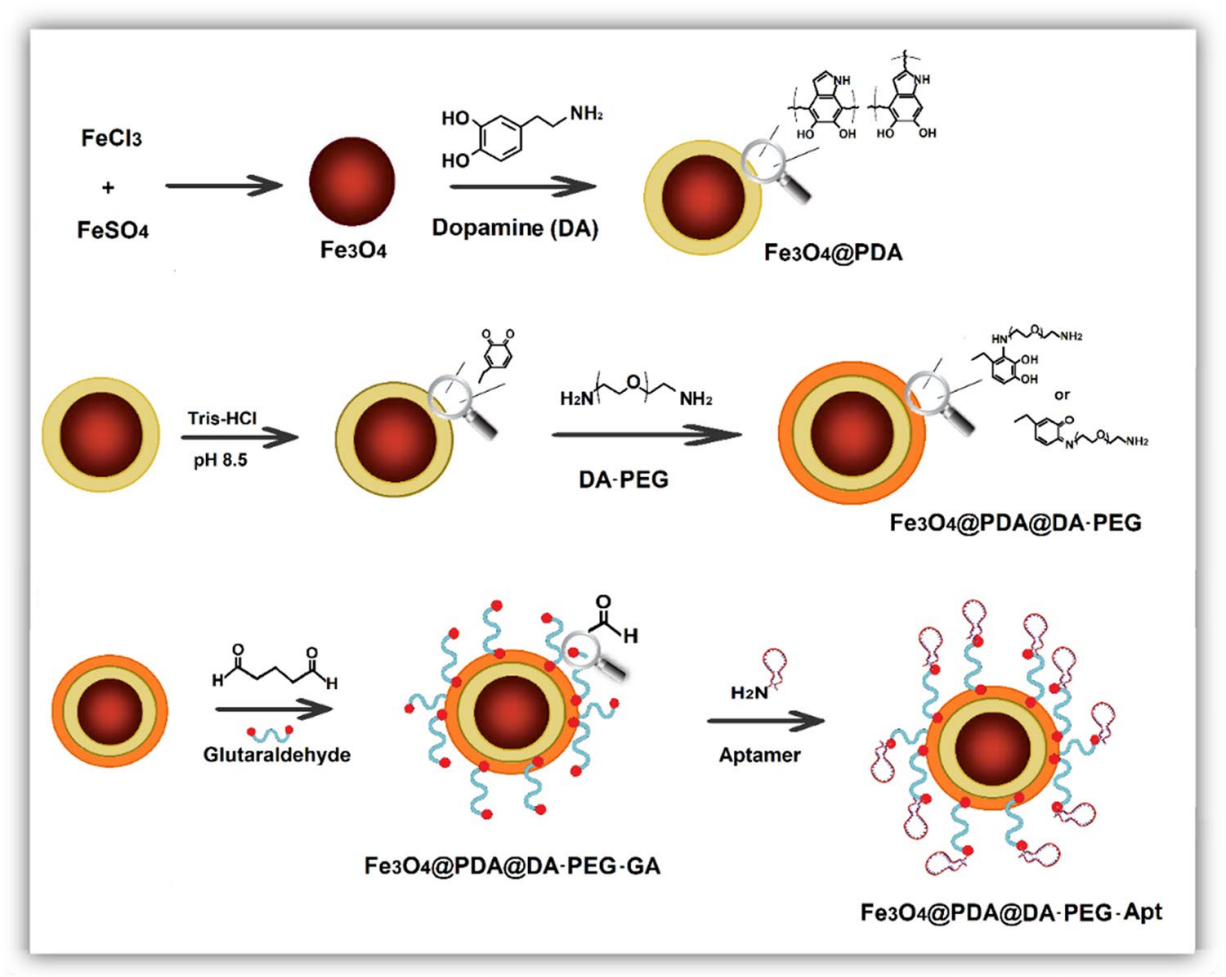
Preparation of the Fe_3_O_4_@PDA@DA-PEG-Apt particles

A novel QCM system was designed for the selective determination of *L. monocytogenes* from food samples. The QCM system was integrated with a magnetic pre-enrichment system with the same *L. monocytogenes*–specific aptamer as in the QCM system. The magnetic system provided easy separation of the target bacterium from the sample with a small size adsorbent requirement and a low volume of eluent. The magnetic particles and QCM chips were sequentially grafted with PDA and DA-PED, and *L. monocytogenes*–specific aptamer was immobilized on both after glutaraldehyde activation. The PEG segment of the DA-PEG has antifouling properties and prevents undesired interaction of non-target particles within the samples. The PDA can be easily grafted on the magnetic nanoparticles by simply incubating in an alkaline dopamine solution, and the coating takes place by self-polymerization of dopamine on the magnetic particle surfaces. Then, DAPEG was directly grafted as a second hydrophilic layer to prevent nonspecific adsorption of any impurities in the medium. The DAPEG coating was spontaneously realized via Schiff base reaction of the amine of DAPEG with PDA without an activating agent. The preparation of Fe_3_O_4_@PD@DA-PEG-Apt particles is presented in Fig. 1. The synthesis and partial characterization of double-layer polymer-coated magnetic particles have been reported in a previous study [51].

The equilibrium water content of Fe_3_O_4_@PDA and Fe_3_O_4_@PDA@DA-PEG particles was determined as 24% and 82%, respectively. The water uptake capacity of the Fe_3_O_4_@PDA@DA-PEG particles increased compared to Fe_3_O_4_@PDA particles by about 3.42 upon coating with DA-PEG. It could be due to the addition of the DA-PEG unit because more water molecules have interacted with the PEG segments of the particles. Similar observations have been reported in earlier works [51, 52].

The size distribution of dried Fe_3_O_4_@PDA@DAPEG particles was sieved with a sieve shaker using molecular sieves and determined by weighing the particles of each size. It was determined that the maximum amount of particles was obtained within the range of 150–212-µm size distribution for Fe_3_O_4_@PDA@DAPEG particles. The particles in the 50–212-µm range were also analyzed using a dynamic light scattering instrument (DLS-Nano ZS, Zetasizer; Malvern Instrument Ltd., Malvern, UK). The particle size distribution of Fe_3_O_4_@PDA@DAPEG particles ranged from 152.4 to 211.8 µm, and the average particle size was determined to be 178.9 µm. Therefore, Fe_3_O_4_@PDA@DAPEG particles in the size fraction of 150–212 µm were used to immobilize *L. monocytogenes*–specific aptamer after activation of glutaraldehyde.

Thermal gravimetric TG/DTA data of Fe_3_O_4_, Fe_3_O_4_@PDA, and Fe_3_O_4_@PDA@DAPEG particles are shown in Figure S1. It was observed that the Fe3O4 weight loss was approximately 4.9% up to 240 °C. This mass loss was due to absorbed water molecules and/or small volatile molecules. As observed from Figure S1, the DTA curve was completed with an endothermic peak. Mass loss was found to be 8.9% for Fe_3_O_4_ between 300 and 800 °C and could have resulted from increased decomposition of magnetic particles. The two-stage weight loss observed for Fe_3_O_4_@PDA and Fe_3_O_4_@PDA@DAPEG particles could be due to the loss of physically bound water and/or volatile substances in the low-temperature range, and the mass loss was found to be 6.7 and 8.2%, respectively. Rapid mass loss changes of approximately 28.3% and 34.6% were observed between 260 and 460 °C, respectively, and this could be due to the removal of grafted PDA and DAPEG macromolecules from the structure.

**Fig. 2:**
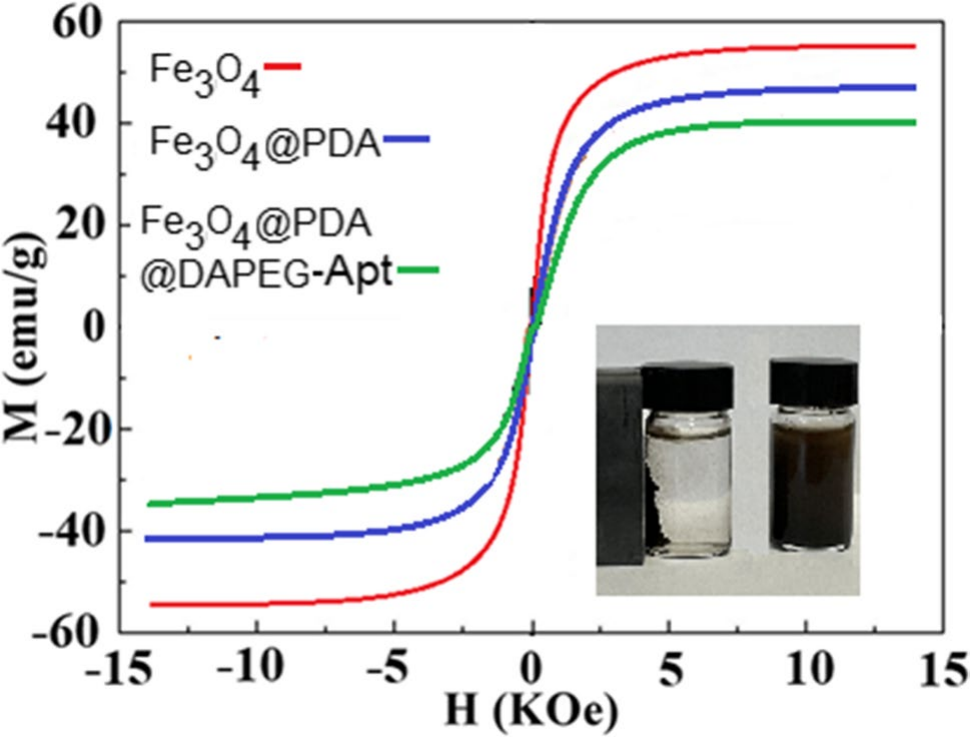
The VSM analysis for magnetic nanoparticles and Fe_3_O_4_, Fe_3_O_4_@PDA, Fe_3_O_4_@PDA@DAPEG-Apt, and magnetic separation of sample from medium using an external magnet

The magnetic properties of the Fe_3_O_4_, Fe_3_O_4_@PDA, and Fe_3_O_4_@PDA@DAPEG were analyzed using VSM at room temperature, and the magnetization curves of the magnetic particles are shown in Fig. 2. The saturation magnetization was determined as 57.2, 40.8, and 36.4 emu/g for Fe_3_O_4_, Fe_3_O_4_@PDA, and Fe_3_O_4_@PDA@DAPEG, respectively. It was observed that saturation magnetization curves of the materials were strongly dependent on the total mass content of Fe_3_O_4_ in the particle and directly affected the magnetic properties of the materials. As can be seen in Fig. 2, the magnetization curves of the samples displayed zero diffraction tendency, exhibiting superparamagnetic properties. These results showed that the prepared Fe_3_O_4_, Fe_3_O_4_@PDA, and Fe_3_O_4_@PDA@DAPEG particles could be easily separated from the solution using an external magnet.

The grafted DAPEG units with pendant amine groups were used for the immobilization of amine groups labeled aptamer specific for *L*. *monocytogenes* cells after glutaraldehyde activation. The immobilization mechanism of L. *monocytogenes* aptamer on the Fe_3_O_4_@PDA@DAPEG is shown in Fig. 1. The amounts of the immobilized aptamers specific for *L*. *monocytogenes* were 5834 µg/g from the 500 ng/mL aptamer initial concentration.

### Immobilization of the selected aptamer on the magnetic particles and chip surface

The cell-SELEX methods have been used to select aptamer sequences with high specificity to the target pathogen microorganisms [13, 18]. Here, aptamer candidates were selected via the SELEX method, as reported previously [18]. The selected aptamer motif has the following sequence, “AGAAATATGCCACCACGTTACAC,” and this sequence is determined to be a candidate that is the most bound to *L. monocytogenes* whole cells compared to other selected candidates. This selected aptamer sequence for *L. monocytogenes* showed high affinity and specificity to the target bacterial cell and was used in the remaining studies.

*L. monocytogenes*–specific aptamer was immobilized on the Fe_3_O_4_@PDA@DA-PEG particles using the bi-functional activating agent glutaraldehyde. The Schiff base reaction was realized between aldehyde groups of the Fe_3_O_4_@PDA@DA-PEG and amine groups of the aptamer ligand. The amount of immobilized aptamer on the magnetic particles was obtained as 5834 µg/g using 500 ng Apt/ mL. Then, the Fe_3_O_4_@PDA@DAPEG-Apt particles were used for the enrichment and determination of the *L. monocytogenes* cells. This magnetic-specific affinity system offered a reasonable recovery of target bacterium from food samples. The Fe_3_O_4_@PDA@DA-PEG-Apt particles provided selective isolation of *L. monocytogenes* from the bacteria-spiked media and food samples with high recoveries between 82.5 and 91.8% using 2.0 mg of the Fe_3_O_4_@PDA@DAPEG-Apt.

**Fig. 3:**
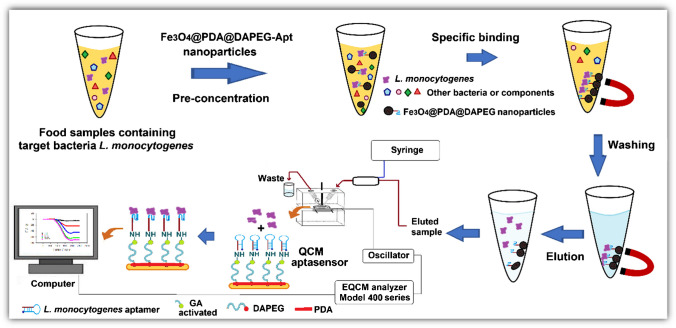
Construction of QCM aptasensor and general schematic representation of the determination of *Listeria monocytogenes* from food samples

The preparation of aptamer immobilized QCM chip for specific and quantitative determination of *L. monocytogenes* cells is presented in Fig. 3. The QCM signals obtained during the preparation of the sensor chips are presented in Table 1. The frequency change of the QCM chip was recorded by incubating it in a dopamine solution. In this way, a self-assembled PDA layer was formed on the QCM-chip surface without any activation agent. The second polymer layer (i.e., DA-PEG) was grafted on the PDA layer; thus, the chip surface gained antifouling properties by incorporating the PEG segment, and the change in the frequency shift after DA-PED coating was recorded (Table S1). In the next step, the aptamer specific for *L. monocytogenes* was immobilized after activation with glutaraldehyde. For the optimization of the immobilized aptamer amounts on the QCM chip surfaces, the concentration of the aptamer in the immobilization medium was changed between 10 and 1000 nmol/L. The QCM chips incubated in the medium about for 6 h, and the frequency changes were measured after washing with PBS after each test. As seen from Table S1, when the aptamer concentration was increased from 10 to 100 nmol/L, the amount of immobilized aptamer increased from 1.42 to 15.28 µg/cm^2^. At concentrations of 100 nmol/L and above, the amount of immobilized aptamer remained at a minimum level. Based on these results, it was decided to use 100 nmol/L aptamer concentration in the remaining experiments.

**Table 1:** Amounts of molecules coated during the preparation of the QCM aptasensor surface

	Hz	∆F^*^	µg cm^−2^	nmol
Au (chip)	−446.60	0	0	0
PDA	−1000.00	553.40	9.78	54.32
DAPEG	−1600.00	600.00	10.60	5.30
GA	−1835.35	235.35	4.16	23.10
Aptamer	−2700.00	864.65	15.28	84.87

^*^Net frequency change obtained after each coating

### Determination of the contact angle values and surface free energy of the prepared chips

Contact angles of gold-coated quartz were determined after each modification protocol. Chip, Chip@PDA, Chip@PDA@DAPEG, and Chip@PDA@DAPEG-Apt surface contact angle values were determined using different test liquids (water, glycerol, and diiodomethane) (Table S2). In addition, the surface contact angle changes before and after the *L. monocytogenes* bacteria attached to the Chip@PDA@DAPEG-Apt sensor were determined using these test liquids, and the relevant surface energy parameters were calculated from these measurements and presented in Table S2. Contact angle values were determined by the standing drop method, at 25 °C, using a digital optical contact angle meter Phoenix 150 (Surface Electro-Optics, Korea) as described previously [53]. According to Young’s equation, the measured contact angle value is smaller by using the test liquids with smaller surface tension. Accordingly, water gave the highest contact angle for all sensor surfaces, and diiodomethane gave the lowest contact angle. As seen in Table S2, coating the chip surface with PDA and DA-PEG caused the contact angles of water to decrease. Meanwhile, the contact angles measured with diiodomethane, which is used as a hydrophobic liquid, increased. Accordingly, it has been observed that the hydrophilicity of the chip surface increases when hydrophilic functional groups are added to the chip surface as a result of modifying the chip surface by coating it with two different polymer layers.

The free surface energy parameters of the chip surface after each coating layer were calculated using the contact angle values of the investigated test liquids (Table S3). The total surface free energies (ɣ^T^) of the Chip, Chip@PDA, Chip@PDA@DAPEG, and Chip@PDA@DAPEG-Apt were calculated using the van Oss method. The results obtained from the van Oss (acid-base) method provide more useful information compared to the Fowkes method [51, 53]. Table S3 shows differences in the total surface energies (ɣ^TOT^) and acid-base components (ɣ^AB^) obtained according to the van Oss method. The PDA and DA-PEG coating or aptamer immobilization on the chip surfaces caused a decrease in water contact angle values and increase in the ɣ^AB^ values and their components. In addition, the Lifshitz-van der Waals component (ɣ^LW^) tends to decrease slightly with the formation of hydrophilic groups on the chip surface. However, while the decrease in the ɣ^LW^ component was at very low levels, it was observed that the increase in the acid-base component (ɣ^AB^) was very high. It can be seen in Table S3 the dispersive components of the samples were larger than the acid-base components. Similarly, the base components (ɣ^−^) were higher than those of the acid components (ɣ^+^) of the chip surface after each coating step. Thus, different contact angle values and different surface energy parameters were obtained after each modification step, showing that the modification steps were carried out successfully.

### Optimization of QCM aptasensor for real-time detection of *L. monocytogenes*

**Fig. 4:**
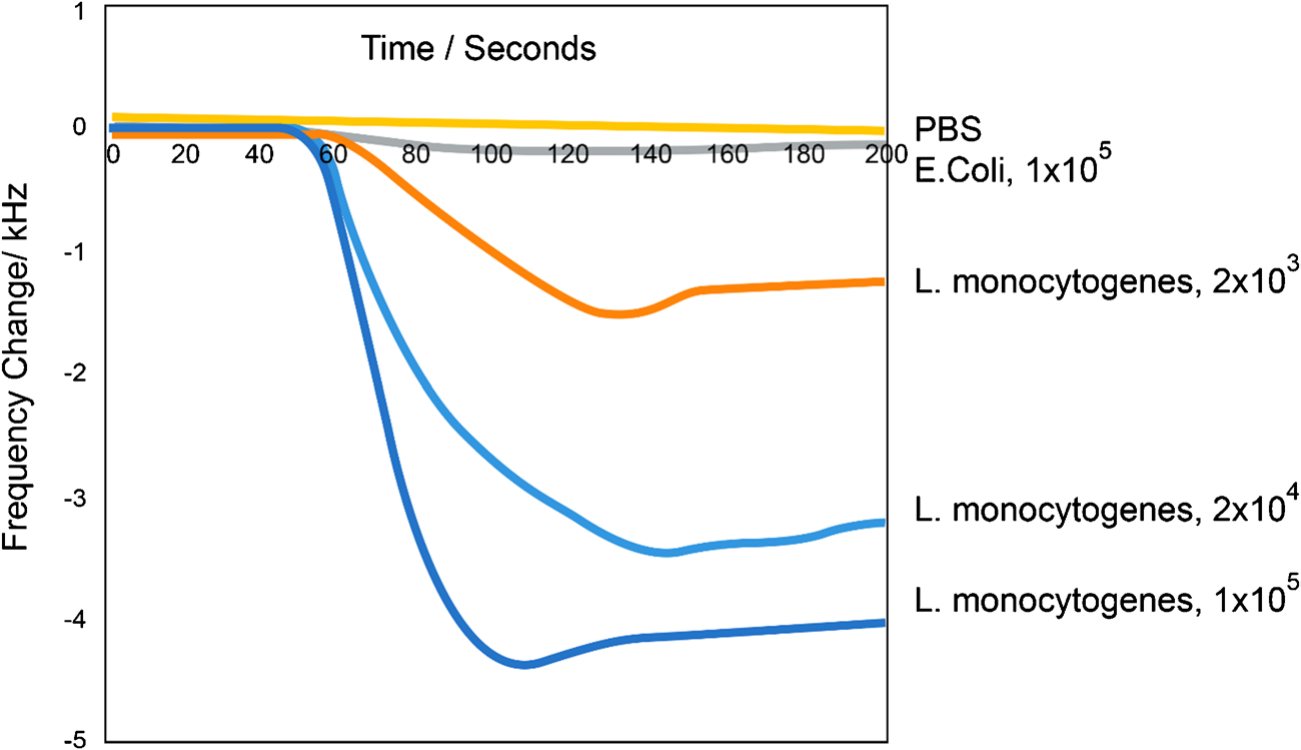
Injection of samples containing *L. monocytogenes* at three different concentration (i.e., 2.0 × 10^3^, 2.0 × 10^4^, 1.0 × 10^5^ CFU/mL) and *E. coli* cells (1.0 × 10^5^ CFU/mL) to the QCM aptasensor

​The selected *L. monocytogenes* binding aptamer was immobilized on the two hydrophilic polymer–grafted QCM chip surface. The immobilized aptamer on the chip surface specifically interacted with the *L. monocytogenes* cells. In addition, the highly polar PEG segment of the DA-PEG improves the hydrophilicity of the microenvironment on the chip surface, and the immobilized aptamer could allow it to interact selectively with the target bacterial cells. Moreover, the selectivity of the aptamer may increase by the increment of the antifouling properties of the chip surface, and the interaction of the impurities with immobilized aptamer could be prevented. The prepared Chip@PDA@DAPEG-Apt was placed in the QCM flow cell holder so that only one side of the chip was in contact with the sample solution in the flow cell. *L. monocytogenes* cells were prepared in PBS at three different concentrations (1.0 × 10^3^, 1.0 × 10^4^, and 1.0 × 10^5^ CFU/mL), and *E. coli* cells (1.0 × 10^5^ CFU/mL) passed through the QCM flow cell at a flow rate of 1000 µL/min by means of a peristaltic pump, and the frequency change in the QCM aptasensor was recorded. The results obtained with three different concentrations of *L. monocytogenes* cells are presented in Fig. 4. Thus, the amount of selectively bonded cell mass on the QCM aptamer surface is quantified in real time, and the mass changes can be easily followed. Moreover, it can quantify in real time how bacteria cells are added or removed from the QCM aptasensor chip. As observed from Fig. 4, the QCM aptasensor frequency change increased as the amount of *L. monocytogenes* cells in the PBS solution increased from 2.0 × 10^3^ to 1.0 × 10^5^ CFU/mL. The change in QCM aptasensor response was proportional to the amount of spiked target cells in the PBS solution, and the total frequency changes were raised along with the amount of spiked bacterial cells in the PBS solution. To detect the binding behavior of the QCM aptasensor to *L. monocytogenes* cells, a control experiment with the non-target eluted *E. coli* cells was done. The amount of introduced cells to the QCM aptasensor was selected as 1.0 × 10^5^ CFU/mL. The obtained frequency change was insignificant and nearly the same as the blank PBS solution. These results showed that other bacterial species did not interact with the selected aptamer or produce a notable response in the QCM aptasensor. These results showed that the presented QCM aptasensor selectively interacted with the *L. monocytogenes* cells.

**Fig. 5:**
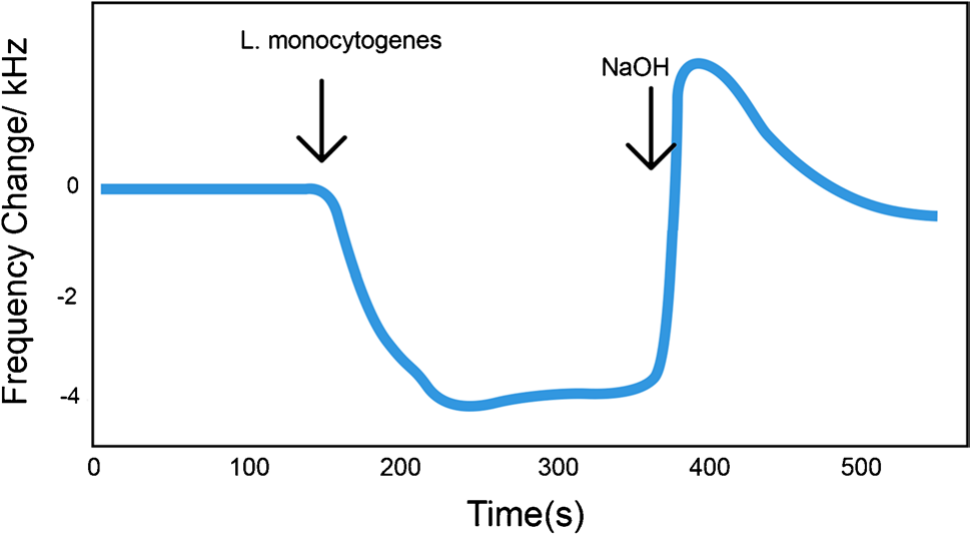
Injection of a sample containing 1.0 × 10^5^ *L. monocytogenes* cells into the prepared QCM aptasensor and regeneration with NaOH

The reusability of the QCM aptasensor mainly depends on overcoming the aptamer-target cell interaction. The hydrogen bonding, hydrophobic, and van der Waals forces between the aptamer and its specific target substrate can be broken by changing salt concentration, medium pH, and adding detergent [50]. In the presented work, regeneration of the aptasensor was realized by applying 20 mmol/L NaOH solution containing 1.0% sodium dodecyl sulfate (SDS). Figure 5 shows *L. monocytogenes* cells of 1.0 × 10^5^ CFU/mL in PBS solution were first introduced to the QCM aptasensor, and the response was recorded. It was observed that the QCM frequency decreased as the target bacterial cells were captured by the QCM aptasensor and continued at a stable frequency level until saturation value was reached. Then, the desorption of *L. monocytogenes* cells was realized by introducing 20 mM NaOH + 1.0% SDS solution. As depicted in Fig. 5, this regeneration mixture was successfully utilized for the breaking of the non-covalent interactions between aptamer and the target bacterial cells, and the bonded bacterial cells were removed from the QCM-aptasensor surface, and thus, the aptasensor was regenerated. These results are in accordance with the earlier reports.

### Fe_3_O_4_@PDA@DAPEG-Apt integrated to QCM aptasensor and detection of

## *L. monocytogenes* from food samples

**Fig. 6:**
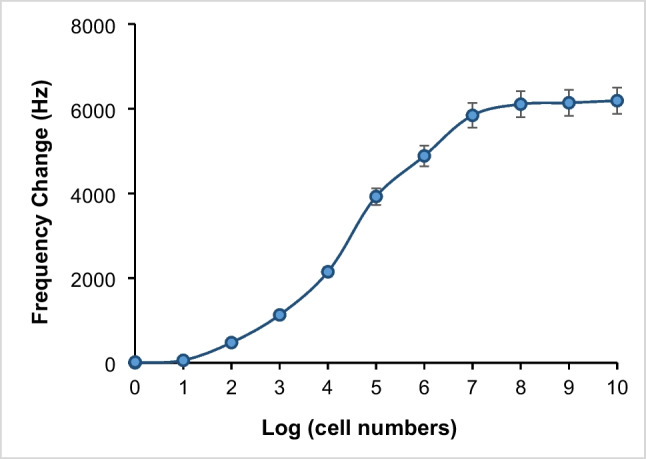
Frequency change obtained against bacterial count after QCM analysis by eluting *L. monocytogenes* samples prepared in PBS buffer after pre-enrichment using 2.0 mg magnetic particles

*L. monocytogenes* binding aptamer was immobilized on the surface of the Fe_3_O_4_@PDA@DAPEG particles and used for the fast capture and rapid elution of *L. monocytogenes* from milk-containing samples. Thus, any existing impurities samples were effectively removed by the presented magnetic pre-enrichment system, as demonstrated by the QCM-aptasensor results. Using the magnetic pre-enrichment capturing of the target bacterium was an appropriate strategy for the real-time detection of the target bacterium in the samples. The binding experiments using QCM aptasensor were achieved to determine the limit of detection (LOD) and limit of quantitation (LOQ) for living *L. monocytogenes* cells. For this, the milk-containing samples spiked with different numbers of *L. monocytogenes* cells (between 1.0 × 10^1^ and 1.0 × 10^10^ CFU/mL) were prepared, and 0.2 mg/mL Fe_3_O_4_@PDA@DAPEG-Apt particle was added for the pre-enrichment of the target bacterium from the sample. The captured *L. monocytogenes* cells were eluted using 20 mM NaOH +1.0 SDS solution. After washing, the extracted cells were fed into a QCM aptasensor for real-time quantitation of *L. monocytogenes* cells in the samples. As depicted in Fig. 6, the QCM aptasensor frequency changes obtained with the target cells in the range of 1.0 × 10^1^ - 1.0 × 10^10^ CFU/mL) in PBS solution, and the logarithm of the cell numbers in the samples were fitted to a three-parameter sigmoidal logistic curve. An increase in the amount of *L. monocytogenes* cells resulted in an increase in QCM aptasensor response. The frequency change was increased linearly from 1.0 × 10^2^ to 1.0 × 10^7^ and reached a plateau value within 4.0 min (*R* = 0.9990, *R*^2^ = 0.9980, CV=0.5350) (Fig. 6). The limit of detection (LOD) was calculated according to the 3-sigma method:

5 $${\text{LOD}}= 3.3\upsigma /{\text{S}}$$

Here, “σ” is the standard deviation of the response and “S” is the slope of the calibration curve.

The limit of quantitation (LOQ) was calculated using the following formula:

6 $$\mathrm{LOQ }= 10\upsigma /\mathrm{ S}$$

The LOD and LOQ were determined as 148 CFU/mL and 448 CFU/mL, respectively.

The amount of adsorbed bacterial cells at various initial concentrations was detected to calculate adsorption isotherms experimentally. The interaction between *L. monocytogenes* cells and QCM aptasensor chip using Langmuir and Freundlich isotherm models was analyzed by the following equations:

7 $${\text{Langmuir}}:\mathrm{ \Delta f }=\mathrm{ \Delta fmax }[{\text{C}}] / {{\text{K}}}_{{\text{a}}} +\mathrm{ C}$$

8 $${\text{Freundlich}}:\mathrm{ \Delta f }= {\mathrm{\Delta f}}_{{\text{max}}} {[{\text{C}}]}^{1/{\text{n}}}$$

∆fmax and Ka are the frequency change after the binding sites on the surface and the constants of the Langmuir isotherm model, respectively. The Langmuir model proposes that binding takes place in a monolayer on a uniform surface [16, 54–56]. The Freundlich model indicates that binding is not limited to monolayer formation and that binding capacity increases as concentration increases [17, 57, 58]. The K_a_ value and ∆f_max_ values of the target bacterium were calculated from the intercept and slope values of the Langmuir isotherm plots as 1.02 × 10^−6^ (1/cells) and 6190.5 Hz, respectively (Table 2). The *R*^2^ values of the Langmuir and Freundlich isotherm models were found to be 0.999 and 0.880, respectively. Moreover, Δf_max_ value was very close to the experimental value (Table 2). Therefore, the Langmuir model well-described the interaction between target cells and the aptamer on QCM aptasensor surface. All experiments were performed three times with reproducible results.

**Table 2:** Langmuir and Freundlich isotherm model constants and correlation coefficients

Langmuir				Freundlich	
(ΔF)_max_ (Hz)	K_a_ × 10^6^ (1/cell#)	*R*^2^	*n*	*K*_F_	*R*^2^
6190.5	1.02	0.999	4.24	90.1	0.880

### Real-time detection of *L. monocytogenes* from contaminated food samples using the QCM aptasensor

**Fig. 7:**
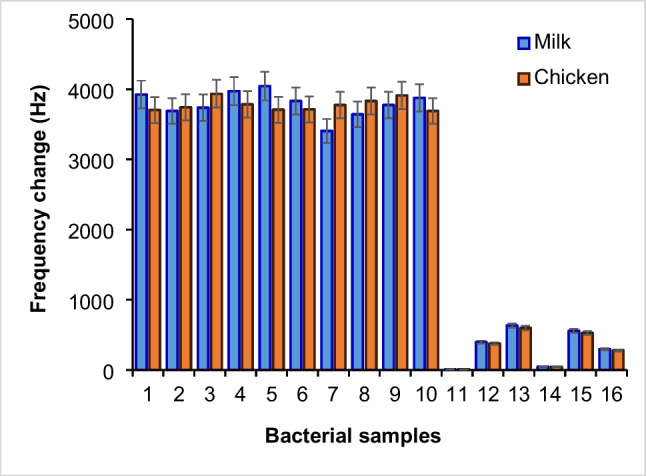
The QCM aptasensor-magnetic pre-enrichment system as applied with various bacteria (10^3^ CFU/ml) in milk (blue) or chicken (orange) matrix. 1, *L. monocytogenes (*ATCC 7644); 2, *L. monocytogenes (raw milk); 3, L. monocytogenes (cream cheese isolate); 4, L. monocytogenes (chicken meat isolate); 5, L. monocytogenes (chicken liver isolate); 6, L. monocytogenes (ground beef isolate); 7, L. monocytogenes (lettuce isolate); 8, L. monocytogenes (cabbage isolate); 9, L. monocytogenes (spinach isolate); 10, L. monocytogenes (curd cheese isolate); 11, E. coli* (ATCC 12435) *12, L. innocua* (ATCC 33090); *13*, *L. ivanovii* (ATCC 19119); *14*, *L. seeligeri* (ATCC 35967); *15, S. aureus* (ATCC 43300); *16, B. subtilis* (garden soil isolate)

Different bacterial isolates grown in culture media containing milk and chicken meat were captured using Fe_3_O_4_@PDA@DAPEG-Apt and eluted from media using 20 mM NaOH + 1.0% SDS. After dilutions of 1.0 × 10^4^ CFU/mL in PBS solution, their frequency values were obtained using QCM aptasensor. As can be seen from Fig. 7, the proportional QCM aptasensor signals were obtained from different concentrations of *L. monocytogenes* preparations. The bacteria from various contaminated food samples were also isolated using a magnetic pre-enrichment system and diluted at a 1.0 × 10^4^ CFU/mL level. Then, all these samples were analyzed using a prepared QCM aptasensor. Furthermore, the number of bacterial cells in the isolates of reference bacteria and food samples was also determined by the plate count method (Table 3). In all these quantitation studies, the aptasensor system worked quite efficiently. As shown in Fig. 7, when compared to the reference *L. monocytogenes* (ATCC 7644) with the other *Listeria strains* (i.e., *L. ivanovii* (ATCC 19119), *L. innocua* (ATCC 33090), and *L. seeligeri* (ATCC 35957)), their QCM signal values significantly decreased up to 10.6%, 16.1%, and 1.2%, respectively. It was also observed that *S. aureus* and *B. subtilis* were gram-positive bacteria like *L. monocytogenes* and had signal values of approximately 14.2% and 7.5% compared to the reference bacteria, whereas *E. coli*, a gram-negative bacterium, produced 0.2% of the signal of the reference strain of *L. monocytogenes*. These results showed that the magnetic pre-enrichment system and QCM-aptasensor were highly selective to the reference bacterium compared to *L. monocytogenes* strains and the other tested bacteria. The selected *L. monocytogenes* binding aptamer was observed to be highly sensitive to other *Listeria* strains in the food samples, as well as to be selective against different types of bacteria. It should be noted that parallel results were obtained for bacteria growing in milk and chicken meat containing mediums. These results showed that the QCM signal values of the bacteria grown and isolated in chicken meat or milk containing medium did not significantly change and gave parallel results. Finally, the reference strain (*L. monocytogenes* ATCC 7644) and various nine food isolates were analyzed using a QCM aptasensor, and the obtained QCM signal values were very close to each other (Fig. 7). Moreover, the developed QCM aptasensor was highly non-sensitive against various gram-negative and gram-positive bacteria.

**Table 3:** QCM aptasensor analysis and plate counting method results of *L. monocytogenes* (7544) at four different concentrations and *L. monocytogenes* samples grown in different food samples

	*Source of L. monocytogenes isolate*	^1^Eluted bacteria (CFU/mL)	^2^Bacteria remaining in the supernatant (CFU/mL)	^3^Initial number of bacterium (CFU)	QCM-aptasensor frequency change (Hz)	Recovery (%)
1	*L. monocytogenes* (7544) (10^2^)	113	24	137	831.6	82.5
2	*L. monocytogenes* (10^3^)	1137	167	1304	2929.9	87.2
3	*L. monocytogenes* (10^4^)	13,696	1235	14,931	3826.2	91.8
4	*L. monocytogenes* (10^6^)	113,906	12598	126,504	5843.7	90.1
5	*L. monocytogenes* (raw milk) (10^4^)	10,099	1632	11,731	3941.9	86.1
6	*L. monocytogenes* (cream cheese) (10^4^)	10,725	1385	12,110	3878.2	88.6
7	*L. monocytogenes* (chicken meat) (10^4^)	10,813	1568	12,381	3897.9	87.3
8	*L. monocytogenes* (chicken liver) (10^4^)	9963	1879	11,842	3903.2	84.1
9	*L. monocytogenes* (*ground beef*) (10^4^)	9957	1797	11,754	3969.5	84.7
10	*L. monocytogenes* (lettuce) (10^4^)	10,596	1345	11,941	3899.0	88.7
11	*L. monocytogenes* (cabbage) (10^4^)	10,107	1430	11,537	3912.4	87.6

^1^Number of desorbed bacteria (CFU/mL) after interaction with Fe_3_O_4_@PDA@DAPEG-Apt particles

^2^Number of residual bacteria (CFU/mL) remaining in the adsorption medium after interaction with Fe_3_O_4_@PDA@DAPEG-Apt particles

^3^The CFU/mL values obtained after plate counting

The correlation of the QCM aptasensor data was realized by colony counting of the tested bacteria after serial dilution according to the plate count method (Maturin and Peeler, 2001). The four different concentrations of *L. monocytogenes* reference strain (1.0 × 10^2^, 1.0 × 10^3^, 1.0 × 10^4^, and 1.0 × 10^6^) and the *L. monocytogenes* isolates from different food samples (such as raw milk, cream cheese, chicken meat and ground beef, lettuce, and cabbage) were determined using plate counting methods given above. For example, approximately 1.0 × 10^4^ bacterial cells were added to PBS solution and contacted with Fe_3_O_4_@PDA@DAPEG-Apt nanoparticles, and the frequency change of the eluent in the QCM aptasensor was found to be 3826.2 Hz. From the eluent plate count analysis, it was found that the number of *L. monocytogenes* cells was found to be13,696 CFU/mL, and the amount of bacteria remaining in the medium after contact was 1235 CFU/mL (Table 3). It was observed that Fe3O4@PDA@DAPEG-Apt particles removed 91.8% of the initial target bacteria in the environment. It is seen that proportional QCM aptasensor signals were obtained from L. monocytogenes at different concentrations (Table 3). Finally, the results were compatible with standard bacteria at a dilution of 1.0 × 10^4^ for various food sample isolates, and it was observed that the sensor system worked quite efficiently. As can be seen from this result, the number of cells in the reference bacterial and food sample isolates prepared in PBS was determined accurately by the plate method and with the help of the QCM-aptasensor integrated into the magnetic pre-enrichment system.

### Competitive adsorption studies

**Fig. 8:**
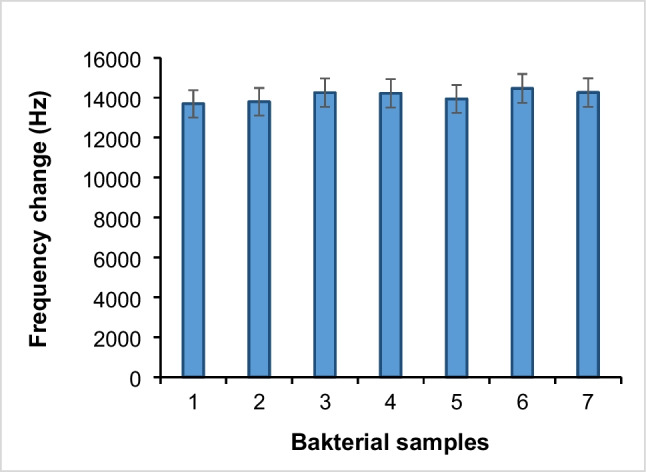
Competitive adsorption of selectivity of the QCM system from concentrations of the order of 1.0 × 10^5^ CFU/mL from binary bacterial mixtures. 1, *L. monocytogenes* (ATCC 7644); 2, *E. coli* (ATCC 12435); 3, *L. innocua* (ATCC 33090); 4, *L. ivanovii* (ATCC 19119); 5, *L. seeligeri* (ATCC 35967); 6, *S. aureus* (ATCC 43300); 7, *B. subtilis* (garden soil isolate). Experimental conditions: volume 2 mL; adsorbent amount 2.0 mg; temperature 25 °C; contact time 30 min. The concentration of each different bacterial species was prepared at 10^4^ levels and 1.0-mL sample was taken and mixed with 1.0 mL *L. monocytogenes* (ATCC 7644) and competitive studies were carried out

As can be seen in Fig. 8, the QCM aptasensor and pre-enrichment systems were quite selective in competitive adsorption studies. The presence of equal amounts of different bacteria in the medium compared to *L. monocytogenes* (ATCC 7644) increased the QCM signal values of the system: 0.74% for *E. coli* (ATCC 12435), 4.06% for *L. innocua* (ATCC 33090), 4.06% for *L. ivanovii* (ATCC 19119), 1.77% for *L. seeligeri* (ATCC 35967), 5.60% for *S. aureus* (ATCC 43300), and 4.10% for *B. subtilis*. As observed, the increase in the signal values was insignificant. It was observed that *S. aureus*, a gram-positive bacterium like *L. monocytogenes*, produced a signal of 5.6%, and *B. subtilis*, another gram-positive soil bacteria, had a 4.1% signal compared to the reference bacterium. In addition, the signal from QCM aptasensor was obtained in the order of 0.74% for *E. coli*, a gram-negative bacterium. These results showed that the developed QCM aptasensor system is highly non-sensitive to the other tested bacteria. Moreover, parallel results showed that the sensitivity of the QCM sensor was not changed in the tested bacteria grown in chicken meat or milk media and gave very similar results (Fig. 8).

### Comparison of the efficiency of the presented QCM aptamer with other methods for the determination of *L. monocytogenes*

Compared with other aptamer-based sensor system for the detection of *L. monocytogenes*, the presented system was noticeably simple and had a high accuracy and specificity. To test the selectivity of the system, three different *Listeria strain*s and three different bacteria species *S. aureus, E. coli,* and *B. subtilis* were selected as alternative microbial cell for the presented QCM aptasensor system. The recently reported aptamer-based sensor systems for detection of *L. monocytogenes* are summarized in Table 4. As can be seen, the presented QCM aptasensor has a wider concentration detection range and comparable detection limit compared with other methods [59–66]. The performance of the QCM aptasensor was mainly attributed to the effective magnetic pre-enrichment capture of the target bacterium using the aptamer-magnetic particles and the sensitivity of the QCM aptasensor system. Moreover, the presented QCM aptamer system has a short time detection properties, and the pre-enrichment method is time-saving. The detection process can be finished within 5.0 min, and the system has great potential in pathogen detection.

**Table 4:** Comparison of the efficiency of the presented QCM-Aptamer with other methods for the determination of *L. monocytogenes*

Analytical methods	Range of detection (CFU/mL)	LOD (CFU/mL)	Reference
Enzyme linked aptasensor with rolling circle amplification	6.1 × 10^3^ to 6.1 × 10^7^	4.6 × 10^2^	[59]
Fluorescence aptasensor	6.8 × 10^1^ to 6.8 × 10^7^	0.8 × 10^1^	[60]
Aptamer-based sandwich assay	2.0 × 10^1^ to 2.0 × 10^6^	2.0 × 10^1^	[61]
Fiber optic biosensor	1.0 × 10^3^ to 1.0 × 10^5^	1.0 × 10^3^	[62]
Aptamer-regulated Pt nanoparticles/hollow carbon spheres	1 × 10^1^ to 1.0 × 10^9^	1.0 × 10^3^	[63]
Electrochemical luminescence sensor	1.5x10^1^ to 1.5 × 10^7^	0.5 × 10^1^	[64]
Colorimetric sensor	10 × 10^1^ to 1.0 × 10^6^	10 × 10^1^	[65]
Photoelectrochemical aptamer sensor	1.3 × 10^1^ to 1.3 × 10^7^	4.5 × 10^1^	[66]
QCM aptasensor	1.0 × 10^2^ to 1.0 × 10^7^	1.4 × 10^2^	In this work

## Conclusion

A novel QCM aptasensor was constructed to selectively determine *L. monocytogenes* from food samples by combining them with a pre-enrichment magnetic system. These magnetic particle systems provided easy separation of the target bacterium from a medium with a small size adsorbent requirement. The saturation magnetization value of the Fe_3_O_4_@PDA@DAPEG particles was 36.4 emu/g. The experimental parameters were studied in detail to invent the best values for the detection of target bacterium from food samples. The target bacterial cells were captured from complex food samples about 10 min. The presented methods suggested a number of benefits, including fast and specific detection of a target bacterium, low cost, simplicity, and easy regeneration with repeated usability. The amount of immobilized aptamer on the Fe_3_O_4_@PDA@DA-PEG-Apt particles was 5834 µg/g. The particles showed high selectivity to *L. monocytogenes,* and up to 91.8% of the spiked was captured from the medium. The aptasensor displayed a very high range detection limit in the range of 1.0 × 10^2^ to 1.0 × 10^7^ CFU/mL. The detected LOD and LOQ values using aptasensor were 148 and 448 CFU/mL, respectively. Finally, the selectivity studies showed that only *L. monocytogenes* was specifically captured by the Fe_3_O_4_@PDA@DAPEG-Apt particles and quantified by the QCM aptasensor system. Using an aptamer-based magnetic separation system with QCM aptasensor resulted in a rapid and sensitive detection of *L. monocytogenes* in chicken meat and milk samples. These results showed that the developed pre-concentration system and QCM aptasensor are highly insensitive to the other bacteria. The prepared QCM aptasensor provided highly sensitive detection system for the contaminated *L. monocytogenes* pathogen in the food samples and presented a real-time bacterial analysis.

### Supplementary Information

Below is the link to the electronic supplementary material.

Supplementary file1 (DOCX 192 KB)

## Data Availability

The authors declare that all the experimental data are available.
