# Supplementary material for: Quartz crystal microbalance–based aptasensor integrated with magnetic pre-concentration system for detection of Listeria monocytogenes in food samples
Source: Mikrochim Acta. 2024 Apr 3;191(5):235. doi: 10.1007/s00604-024-06307-2 (PMC10990998; doi:10.1007/s00604-024-06307-2)
Supplement: Supplementary file 1 — Supplementary file1 (DOCX 192 KB) [file 604_2024_6307_MOESM1_ESM.docx]

**Supplementary File**

**Quartz crystal microbalance based aptasensor integrated with magnetic preconcentration system for detection of *Listeria monocytogenes* in food samples**

.

**Fatma Beyazit^1^, Mehmet Yakup Arica^2^, Ilkay Acikgoz-Erkaya^3^, Cengiz Ozalp^4^ and Gulay Bayramoglu*^2,5^**

^1^Department of Obstetrics and Gynecology, Faculty of Medicine, Çanakkale Onsekiz Mart University, Çanakkale, Turkey

^2^Biochemical Processing and Biomaterial Research Laboratory, Gazi University, 06500 Teknikokullar, Ankara, Turkey

^3^Department of Environmental Science, Faculty of Engineering and Architecture, Ahi Evran University, Kırsehir, Turkey

^4^Department of Medical Biology, School of Medicine, Atilim University, Ankara, Turkey

^5^Department of Chemistry, Faculty of Sciences, Gazi University, 06500 Teknikokullar, Ankara, Turkey

**Table S1.** Amount of aptamer immobilization at different initial aptamer concentrations

| Aptamer (nM) | Hz* | ΔF** | µg/cm^2^ |
| --- | --- | --- | --- |
| 5 | -1915.56 | 80.21 | 1.42 |
| 10 | -2036.73 | 201.38 | 3.56 |
| 50 | -2461.63 | 626.28 | 11.07 |
| 75 | -2588.57 | 753.22 | 13.31 |
| 100 | -2700.00 | 864,65 | 15,28 |
| 250 | -2707.65 | 872.3 | 15.42 |
| 500 | -2706.75 | 871.4 | 15.40 |
| 1000 | -2705.85 | 870.5 | 15.38 |

**Tablo S2.** Surface contact angles values of the aptasensor chip surfaces

|  | Water  ɣ_l_ = 71.3 | Glyserol  ɣ_l_ = 64.0 | Diiodomethane  ɣ_l_ = 50.8 |
| --- | --- | --- | --- |
|  | θ (°) | θ (°) | θ (°) |
| Chip | 64.4 | 59.6 | 33.1 |
| Chip@PDA | 57.2 | 49.3 | 45.1 |
| Chip@PDA@DAPEG | 50.9 | 65.2 | 34.3 |
| Chip@PDA@DAPEG-Apt | 65.8 | 52.3 | 36.5 |
| Chip@PDA@DAPEG-Apt-*L.Monocytogenes* | 78.6 | 63.4 | 24.7 |

**Tablo S3.** Surface free energy parameters of Chip, Chip@PDA, Chip@PDA@DAPEG and Chip@PDA@DAPEG-Apt sensor according to the van Oss method

|  | ɣ^LW^  (mN/m^2^) | ɣ^+^  (mN/m^2^) | ɣ^-^  (mN/m^2^) | ɣ^AB^  (mN/m^2^) | ɣ^TOT^  (mN/m^2^) |
| --- | --- | --- | --- | --- | --- |
| Chip | 43.6 | 0.28 | 3.37 | 2.19 | 45.8 |
| Chip@PDA | 36.2 | 1.81 | 3.40 | 10.5 | 46.7 |
| Chip@PDA@DAPEG | 42.2 | 0.59 | 6.12 | 8.24 | 50.4 |
| Chip@PDA@DAPEG-Apt | 45.6 | 0.52 | 1.81 | 1.70 | 47.3 |
| Chip@PDA@DAPEG-Apt-*L. monocytogenes* | 41.1 | 0.52 | 1.73 | 1.81 | 42.9 |


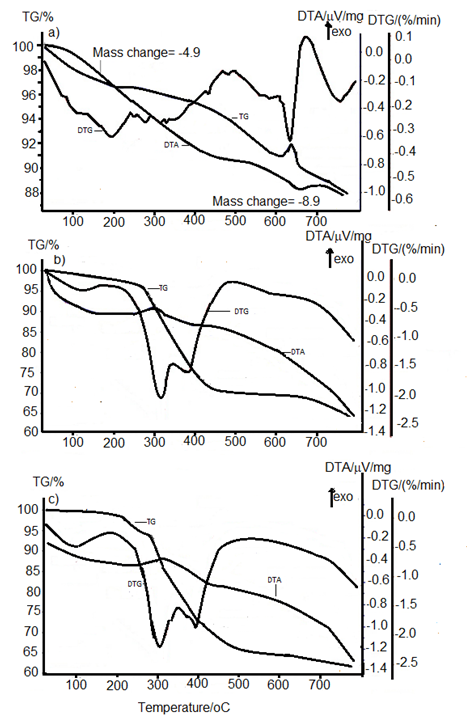


**Figure S1.** TG, DTG and DTA analysis of the Fe_3_O_4_ (A), Fe_3_O_4_@PDA (B) Fe_3_O_4_@PDA@DAPEG, and (C) Fe_3_O_4_@PDA@DAPEG particles.
